# Biomarkers in Urachal Cancer and Adenocarcinomas in the Bladder: A Comprehensive Review Supplemented by Own Data

**DOI:** 10.1155/2018/7308168

**Published:** 2018-03-12

**Authors:** Henning Reis, Ulrich Krafft, Christian Niedworok, Orsolya Módos, Thomas Herold, Mark Behrendt, Hikmat Al-Ahmadie, Boris Hadaschik, Peter Nyirady, Tibor Szarvas

**Affiliations:** ^1^Institute of Pathology, University Hospital Essen, University of Duisburg-Essen, Hufelandstr 55, 45147 Essen, Germany; ^2^Department of Urology, University Hospital Essen, University of Duisburg-Essen, Hufelandstr 55, 45147 Essen, Germany; ^3^Department of Urology, Semmelweis University, Üllői út 78/b, 1082 Budapest, Hungary; ^4^Department of Urology, The Netherlands Cancer Institute - Antoni van Leeuwenhoek Hospital, Plesmanlaan 121, 1066 CX Amsterdam, Netherlands; ^5^Department of Pathology, Memorial Sloan Kettering Cancer Center, 1275 York Avenue, New York, NY 10065, USA

## Abstract

Urachal cancer (UrC) is a rare but aggressive cancer. Due to overlapping histomorphology, discrimination of urachal from primary bladder adenocarcinomas (PBAC) and adenocarcinomas secondarily involving the bladder (particularly colorectal adenocarcinomas, CRC) can be challenging. Therefore, we aimed to give an overview of helpful (immunohistochemical) biomarkers and clinicopathological factors in addition to survival analyses and included institutional data from 12 urachal adenocarcinomas. A PubMed search yielded 319 suitable studies since 1930 in the English literature with 1984 cases of UrC including 1834 adenocarcinomas (92%) and 150 nonadenocarcinomas (8%). UrC was more common in men (63%), showed a median age at diagnosis of 50.8 years and a median tumor size of 6.0 cm. No associations were noted for overall survival and progression-free survival (PFS) and clinicopathological factors beside a favorable PFS in male patients (*p* = 0.047). The immunohistochemical markers found to be potentially helpful in the differential diagnostic situation are AMACR and CK34*β*E12 (UrC versus CRC and PBAC), CK7, *β*-Catenin and CD15 (UrC and PBAC versus CRC), and CEA and GATA3 (UrC and CRC versus PBAC). Serum markers like CEA, CA19-9 and CA125 might additionally be useful in the follow-up and monitoring of UrC.

## 1. Introduction

The urachus is a remnant of the fetal structure connecting the allantois and the fetal bladder. During early fetal development, the urachus usually regresses to form an obliterated fibromuscular canal, known as the median umbilical ligament [1–4]. Failure of complete luminal obliteration has been described in up to one-third of adults and can rarely lead to various anomalies including cysts, fistulas, and diverticula or rarely malignant transformation [2, 5].

Our understanding of urachal cancer (UrC) has evolved since the seminal studies by Begg [6] in the 1930's following the first report by Hue and Jacquin [7] in 1863 and earlier works of Cullen in 1916 [8]. UrC is a very rare but highly malignant tumor entity with an incidence of <1% of all bladder cancers [1, 9, 10]. Establishing the diagnosis of UrC can be challenging for the urologist, pathologist, and radiologist and usually requires a multidisciplinary approach. In terms of histopathology, many overlapping features with the main differential diagnostic entities exist. While the diagnostic criteria adapted and established by Sheldon et al. [11] are most widely used, Gopalan and colleagues [1] modified these criteria and Paner and colleagues proposed diagnostic criteria for nonglandular type UrC [12].

Recently, Paner and colleagues also gave a review on the diagnosis and classification of urachal epithelial neoplasms [13]. To furthermore give a current overview of the clinical and therapeutical implications of UrC, we have recently conducted a meta-analysis of the literature including 1010 cases of UrC [14].

Histologically, urachal adenocarcinomas (which are the most common carcinomas of urachal origin) overlap with their main differential diagnostic entities, that is, primary bladder adenocarcinomas and colorectal adenocarcinomas. The present work therefore aims to provide an overview and summary of the immunohistochemical biomarkers in UrC and their potential role in the diagnosis of such tumors. It is combined with clinicopathological evidence and its data is collected from the published literature since 1930. Additionally, it is supported by our own data of immunohistochemical expression in 12 UrC cases with 11 different antibodies including the report of GATA3 expression in this disease.

## 2. Literature Review and Statistics

**Figure 1:**
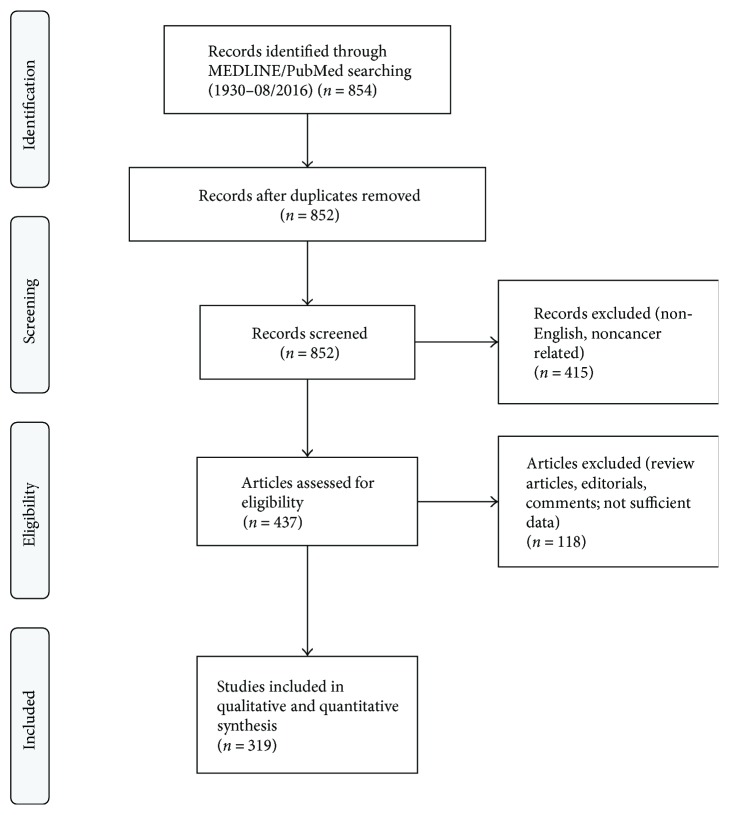
PRISMA flow diagram. The diagram illustrates the phases and selection criteria used for study selection in this work.

A PubMed search was conducted using the string [“urachus carcinoma” OR “urachus cancer” OR “urachal carcinoma” OR “urachal cancer”] which returned 854 results (end of data acquisition: 08/2016). The algorithm of study selection is illustrated in Figure 1. Information was extracted from whole papers written in English language and from English abstracts in case of other primary language. In case of different entities in the papers, only information regarding UrC was extracted. When available, survival data was recorded for both overall survival (OS) and progression-free survival (PFS). For statistical analyses, SPSS (v23; IBM, Armonk, USA) was used. Pearson correlation analysis was conducted when appropriate. Survival analyses were conducted using the Kaplan Meier method with the log-rank test and univariable Cox analysis. When appropriate, continuous variables were dichotomized at their median level for analysis of their impact on survival.

## 3. Additional Data from Our Own Institution

The clinicopathological data of our cohort has been published previously [15]. Immunohistochemical studies were performed on formalin-fixed and paraffin-embedded urachal adenocarcinoma tissue using a BenchMark ULTRA System (Ventana Medical Systems, Tucson, USA) following manufacturer's instructions. A total of 11 different antibodies were performed on 12 cases of urachal adenocarcinomas from the University Hospital of Essen including *β*-Catenin, CD15, CDX2, CEA, CK7, CK20, GATA3, MLH1, MSH2, MSH6, and PMS2 (Supplementary Table 1). The study was approved by the ethic committee of the University Hospital of Essen (16-6902-BO, 04.28.2016).

Further details on used antibodies, protocol information, and results of the immunohistochemical analyses are displayed in Supplementary Table 1.

## 4. General Results of the Literature Review

Three hundred and nineteen studies were identified that contained sufficient information on cases of UrC. The number of publications has increased rapidly in the recent years with 169 (53%) publications since the year 2000 and 75 (24%) studies from 2011–2016.

A total of 2154 cases of UrC were identified, with information on UrC histology available in 1984 cases (92%), of which 1834 (92%) were adenocarcinomas. The majority of studies with information on UrC cases were case reports (74%), while contributing only a minor part to the total number of cases (16%). In 1491 cases, gender information was available with evidence showing that most UrC cases occurred in men (63%) compared to women (37%). The mean and median age were 48.6 and 50.8 years, respectively (range: 0.3–86.0 years), and tumor size 7.1 cm and 6.0 cm, respectively (range: 0.5–25.0 cm). Data on tumor grades were sporadic and inconsistent and could not be further analyzed. Survival data were available in 76 cases (adenocarcinomas: *n* = 60, nonadenocarcinomas: *n* = 16) with a median follow-up of 12 months in the total cohort (range: 1–62 months). The median OS for the entire cohort was 46.8 months (adenocarcinomas: 42.7 months) with a 1-year survival of 86% (adenocarcinomas: 86%), 3-year survival of 63% (adenocarcinomas: 59%), and a 5-year survival of 41% (adenocarcinomas: 35%). The median PFS for the entire cohort was 46.6 months (adenocarcinomas: 41.1 months) and 75% at 1-year (adenocarcinomas: 72%), 60% at 3 years (adenocarcinomas: 55%), and 39% at 5 years (adenocarcinomas: 33%). It is important to note that some of the survival data derives from older papers with different treatment strategies that might have affected the outcome analysis. In fact, recent epidemiological studies demonstrate higher survival rates (5-year overall survival of approximately 50%) due to advances in the surgical and medical management of this disease [16].

Detailed clinicopathological data are listed in Table 1. Data on UrC adenocarcinomas were collected from these references [1, 3, 4, 9–11, 15–251].

## 5. Specific Review Data: Adenocarcinomas

Histopathologically, both primary adenocarcinomas of the bladder and urachal adenocarcinomas show similar subtypes although their distribution differs [21, 252, 253]. In invasive urachal adenocarcinoma, the following four subtypes are described in the 2016 WHO classification: *mucinous* (colloidal) type with preponderance of extracellular mucin and malignant epithelia floating within, *enteric (intestinal)* type with preponderance of malignant stratified epithelium resembling colorectal adenocarcinomas, *mixed* type with neither a mucinous nor an enteric pattern prevailing, *not other specified (NOS)* type with a pattern not easily identifiable as mucinous or enteric type, and *signet ring cell* type with signet ring cell morphology prevailing.

It is important to note that the concept of mucinous cystic tumors as recently proposed by Amin et al. [17] was not applied for this study due to the lack of such a classification in the older literature. In their work, Amin et al. described a distinct subgroup of urachal neoplasms with predominant cystic appearance in analogy to similar neoplasms in the ovary. This includes mucinous cystadenomas, mucinous cystic tumor of low malignant potential (MCTLMP), and mucinous cystadenocarcinoma with microinvasion or frank invasion. However, although in cox analyses tumor size was not associated with survival, not larger but smaller tumor size exhibited a higher hazard ratio for OS possibly giving support to the concept of favorable prognosis of mucinous cystic lesions of urachal origin [17].

**Figure 2:**
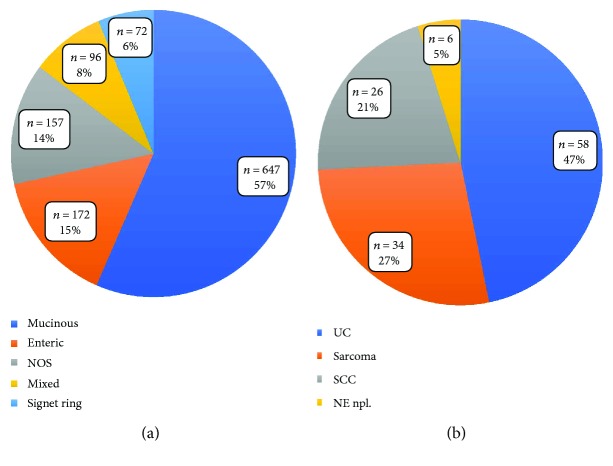
Distribution of the different types of UrC (a) in urachal adenocarcinomas with available information of special type and (b) in nonadenocarcinoma UrC with information of special type. UrC: urachal cancer; NOS; not otherwise specified; UC: urothelial carcinoma; SCC: squamous cell carcinoma; NE npl.: neuroendocrine neoplasms.

In a population-based study from Wright and colleagues including 151 UrC adenocarcinomas and 1374 primary bladder adenocarcinomas, the mucinous/colloid pattern was detected in 48% of UrC adenocarcinomas [16]. Also in our analysis, the mucinous type represented the most common special type of urachal adenocarcinomas (57%) (Table 1, Figure 2(a)). In Wright and colleagues' work, the second most common pattern was the NOS type (39%), which in our analysis accounted for 14%. However, in their analysis, the enteric type was not explicitly mentioned, which comprised 15% in our study. Additionally, they described the signet ring cell type and the mixed type in 7% each, which comprised 6% and 8% in our analysis, respectively. The prognostic value of these histopathologic features, however, remains to be established while a more favorable clinical course for UrC adenocarcinomas as compared to primary bladder adenocarcinomas was found [16]. Additionally, presence of signet ring cell morphology and higher tumor grade were identified of unfavorable prognostic value in some [10, 21, 157, 192, 221] but not all series [24] of UrC adenocarcinomas. Regarding signet ring cell morphology, this may result from the differing definitions and lack of consistent cut-off on the amount of signet ring cells across the different studies. In our survival analysis from cases of the literature, we could not detect an influence of type of UrC adenocarcinoma on OS, but a borderline influence on PFS in terms of a survival benefit for intestinal type UrC. However, this finding was not consistent in further (Kaplan Meier) analyses, thus preventing further conclusions. Data density was too low for any tumor grading-related analyses.

Regarding the gender distribution, our review data is similar to epidemiological studies with preponderance of male UrC patients (62.8%) [16]. While no significant influence of gender was detectable on OS in our accumulated data from the literature, male gender was associated with improved PFS (*p* = 0.047), an effect which was mainly related to the adenocarcinoma part of the cohort (*p* = 0.009; Supplementary Figure 1). This effect has not yet been mentioned in the literature while its cause remains to be elucidated. It seems not to be related to median tumor size or age at diagnosis as these factors were not associated with gender. Additionally, no significant prognostic associations were noted for these two factors, neither in the total cohort nor in subgroup analyses.

## 6. Specific Review Data: Nonadenocarcinoma Neoplasms

In addition to UrC adenocarcinomas, nonglandular urachal tumors are included in the recent WHO 2016 classification. These are urothelial, squamous, neuroendocrine, and mixed-type neoplasms, which are stated to account for 4% to 27% of cases [12, 13, 253, 254]. These neoplasms are histologically and immunophenotypically similar to their counterparts elsewhere in the body [253].

Our analysis yielded 124 (of 150) cases of nonadenocarcinoma UrC with further classified histology. Urothelial carcinomas (UC) represented the largest group (*n* = 58, 47%) (Table 1, Figure 2(b)) [3, 10–12, 19, 23, 46, 71, 82, 85, 127, 223, 255–267]. The second largest group was the group of sarcomas (*n* = 34, 27%), however, with a large variety of different entities including childhood rhabdomyosarcoma (embryonal, alveolar and NOS types) [268–272], leiomyosarcomas [273–276], fibrosarcomas [277, 278], and also some cases without further specification of the type of entity [3, 11, 47, 279–281]. Attention has to be paid to the fact that the reported sarcomas derive from a broad timespan with different knowledge levels, respectively. Therefore, some of these neoplasms would today be classified differently. In addition to mesenchymal lesions, our analysis showed 26 cases of squamous cell carcinomas (SCC; 21%) [11, 71, 85, 105, 223, 282–291], followed by neuroendocrine neoplasms including small cell carcinomas with 6 cases (5%) [9, 10, 12, 201]. In the remaining cases, information on nonadenocarcinoma entity was at least partly missing [3, 11, 19, 71, 85, 141, 292, 293] or included other entities such as yolk sac tumors [187, 294, 295] or a neuroblastoma [296].

In addition to malignant urachal tumors, several other intermediate and benign tumors or conditions of the urachus have been reported some mimicking urachal cancer and thus posing a differential diagnostic problem. Tumors or conditions rated as intermediate include inflammatory myofibroblastic tumors (IMT) [297–300], a solitary fibrous tumor (SFT) [301], desmoid fibromatoses [302, 303], a hemangiopericytoma [304], and a Castleman's disease [305], while benign tumors and conditions include dermoid cysts [301, 306], teratomas [307, 308], leiomyomas [309, 310], (fibrous) hamartomas [311, 312], a hemangioma [313], a fibroadenoma [314], malakoplakia [315], abscesses [316–318], a xanthogranulomatous urachitis [319], a urachal tuberculosis [320], actinomycosis [321–323], an endometriosis [324], a perforated colonic diverticulitis [325], and even a fishbone within an urachal cyst [326].

## 7. Biomarkers in Urachal Cancer: Immunohistochemistry

Given the extensively overlapping histopathological features of adenocarcinomas of urachal and primary bladder origin on the one hand and secondary adenocarcinomas from different sites on the other, biomarkers for differential diagnostic purposes are required. The most important differential diagnostic problems with significant impact on therapeutic decisions may be categorized as follows:

1. Differentiation between invasion/metastasis of colorectal adenocarcinomas and urachal adenocarcinomas. Exclusion of a possible invasion of this cancer (to the bladder) is a necessary step for the definitive diagnosis of UrC and of therapeutic relevance.
2. Distinguishing urachal adenocarcinomas from those of primary bladder origin has also a direct clinical impact on the surgical treatment. In localized disease, primary bladder adenocarcinomas are usually treated with complete cystectomy while urachal adenocarcinomas mostly require partial cystectomy with *en bloc* removal of the umbilical ligament and umbilicus (radical versus partial cystectomy) with significantly different impact on quality of life [4, 85].
3. Identification of the origin of a (mucinous) adenocarcinoma of unknown primary is also important as urachal adenocarcinomas frequently metastasize to various organs, such as the bone, lung, and liver. Identification of urachal origin of a (mucinous) adenocarcinoma can have a direct therapeutic consequence [4].

**Figure 3:**
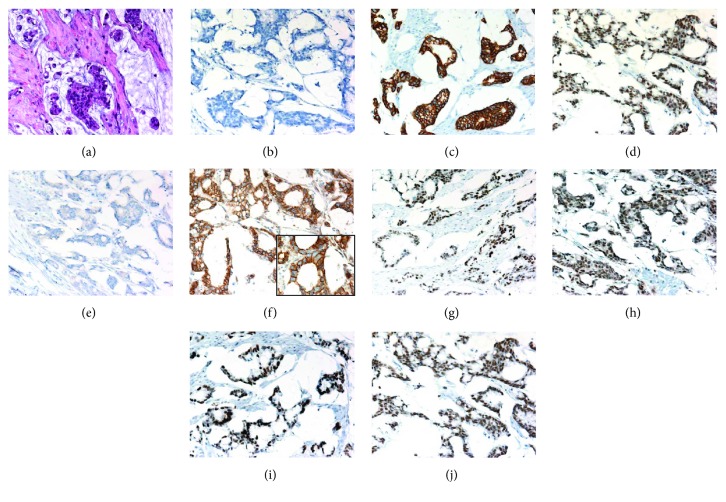
A representative case of mucinous urachal adenocarcinoma. (a) Atypical cells floating in extracellular mucin. Focal signet ring cell morphology is noticeable (H&E staining). The case exhibited a typical profile in further immunohistochemical studies with no reactivity for CK7 (b) but positive reactivity for CK20 (c) and CDX2 (d, nuclear). As in all analyzed cases, no GATA3 reactivity was noted (e). In the *β*-Catenin immunohistochemistry, a strong membranous and cytoplasmic but no nuclear reactivity was noted (f; inlay magnification 600x). The immunohistochemical reactions against the MMR proteins all were positive, that is, MLH1 (g), PMS2 (h), MSH2 (i), and MSH6 (j).

An overview of the immunohistochemical markers assessed in urachal adenocarcinomas is provided in Table 2 and a representative example is illustrated in Figure 3. Further detailed information is provided in Supplementary Table 2.

The immunohistochemical markers most often employed in the work up of adenocarcinomas of different sites usually include Cytokeratin 20 (CK20) and CK7. In our analysis of a total of 116 urachal adenocarcinomas, only 4 cases were negative for CK20—an overall positive rate of 97% [1, 17, 23, 26, 27, 31, 36, 48, 56, 60, 62, 67, 74, 77, 86, 95, 99, 124–126, 128, 148, 156, 163, 164, 216, 217, 220, 240, 245, 251, 327]. Considering the robust CK20 expression in adenocarcinomas of sites of differential diagnostic interest, CK20 has no significant value in this setting.

In contrast, expression of CK7 in these tumors is widely variable. In urachal adenocarcinomas, CK7 exhibited a pooled reactivity rate of 51%, compared to considerable lower rates in colorectal cancer (0–38%, Table 2, Supplementary Table 2) [1, 17, 23, 26, 27, 31, 36, 48, 56, 60, 67, 74, 77, 86, 99, 124, 125, 156, 163, 164, 216, 217, 220, 240, 251, 255, 292, 327, 328]. However, similar to urachal adenocarcinomas, primary bladder adenocarcinomas constantly exhibited relatively high CK7 reactivity rates (33%–70%), thus limiting the value of CK7 in the discrimination between these two entities [23, 329].

Additionally, CK20 and CK7 were the only markers with sufficient data for survival analyses. However, no significant influence of the CK20/CK7 expression profile on OS or PFS was noted.

As a rather specific nuclear marker for intestinal epithelia and corresponding adenocarcinomas, CDX2—a homeobox gene coding for a transcription factor with intestine specificity—has been proposed for differential diagnostic considerations. However, nuclear CDX2 reactivity was evident in the majority of urachal adenocarcinomas (90%) [1, 17, 23, 26, 31, 60, 99, 125, 126, 128, 216, 217, 240, 245, 251, 327, 330] and many primary bladder adenocarcinomas (13%–83%) [329, 331]. In addition to its reactivity in almost all colorectal adenocarcinomas, CDX2 immunopositivity has been detected in considerable numbers in several adenocarcinomas from different sites such as the gastrointestinal tract, pancreas, and ovary [330, 332]. Furthermore, CDX2 reactivity has been described in cystitis glandularis and intestinal metaplasia of the bladder and glandular epithelia of urachal remnants [23, 333–335]. Taken together, CDX2 is not helpful in the differential diagnosis of adenocarcinomas in the urinary bladder.

Another plausible biomarker in this context is *β*-Catenin, a protein involved in cell-cell adhesion and gene transcription regulation [336]. In normal cells, *β*-Catenin staining is restricted to the membrane/cytoplasm, while in colorectal adenocarcinomas, *β*-Catenin exhibits nuclear accumulation due to mutation or loss of the adenomatous polyposis coli (APC) gene then acting as a transcriptional activator [337]. While in colorectal adenocarcinomas nuclear *β*-Catenin expression can be found in the majority of cases, nuclear *β*-Catenin reactivity was detected in a low rate of primary bladder adenocarcinomas (0%–17%) [329, 338]. Similarly, in urachal adenocarcinomas, nuclear *β*-Catenin expression was a rare event. In our summary analysis, any type of nuclear *β*-Catenin was detected in 9 of 63 cases (14%) [1, 17, 23, 26, 125, 216, 220, 328]. APC mutations, however, can be found in urachal adenocarcinomas slightly more often than the immunohistochemical results propose [20, 204]. From a differential diagnostic point of view, nuclear *β*-Catenin expression may be useful in distinguishing primary bladder and urachal adenocarcinomas from secondary bladder involvement by colorectal adenocarcinomas. However, *β*-Catenin is of no use in the differentiation of primary bladder from urachal adenocarcinomas as both entities exhibit comparable *β*-Catenin staining characteristics.

Further potential markers include Claudin-18 and Reg IV, however, with no available data in primary bladder adenocarcinomas. Claudin-18 has been reported to be of diagnostic value especially in pancreatic and gastric cancer, but is rarely expressed in colorectal adenocarcinomas [339–341]. Although exhibiting a positivity rate of 53% in the total number of urachal adenocarcinomas cases, it was found to have only a low positivity rate (27%) in enteric type urachal adenocarcinomas, thus limiting its usefulness in UrC diagnostics with regard to the largest group of intestinal-differentiated colorectal adenocarcinomas [23]. Reg IV is associated with the cellular phenotype of the intestine and expressed in various cancers with intestinal differentiation such as gastric and colorectal cancer [342]. In urachal adenocarcinomas, Reg IV expression was detected in 85% of cases arguing against its potential use in the differential diagnostics of adenocarcinomas detected in the bladder [23]. Both markers additionally failed to demonstrate diagnostic value in signet ring cell UrC compared to signet ring cell carcinoma of colorectal origin [23].

Further, possibly useful biomarkers of urachal adenocarcinomas with data of at least 10 cases are alpha-methylacyl-CoA racemase (AMACR, p504s), CD15 (Leu-M1), carcinoembryonic antigen (CEA), CK34*β*E12 (high-molecular weight cytokeratin), GATA binding protein 3 (GATA3), mucin 2 (MUC2), and mucin 5 AC (MUC5AC).

In urachal adenocarcinomas, AMACR was found to be positive in a low number of cases (17%), while in colorectal and primary bladder adenocarcinomas, a significantly higher number (>66%) of cases exhibited AMACR-reactivity [17, 77, 343, 344]. In contrast, CK34*β*E12 was more frequently positive (67%) in urachal adenocarcinomas, while being variably expressed in primary bladder or colorectal adenocarcinomas [1, 26, 77, 125, 255, 345]. A comparable distribution was detected for MUC2 and MUC5AC with high positivity rates in urachal adenocarcinomas (100% and 92%) and lower rates in colorectal and primary bladder adenocarcinomas, however, with significant overlap [86, 95, 100, 125, 128, 251, 335, 346, 347].

In contrast, CD15 was detected in high rates of both urachal and primary bladder adenocarcinomas (86% and 73%, resp.) compared to colorectal adenocarcinomas with a lower reactivity rate (<50%) [21, 27, 124, 125, 348]. CEA was opposingly found to be positive in all analyzed cases of urachal adenocarcinomas and in a similarly high rate of colorectal adenocarcinomas but lower rates in primary bladder adenocarcinomas (29–67%) [21, 27, 48, 60, 67, 77, 86, 100, 106, 124, 163, 165, 245, 255, 348–353].

Finally, GATA3 was not found to be expressed in urachal adenocarcinomas and colorectal adenocarcinomas but in approximately half of cases of primary bladder adenocarcinomas [335, 354, 355]. In addition, nuclear GATA3 reactivity might be useful in the differential diagnosis of bladder adenocarcinomas with signet ring morphology [355].

The example of GATA3 in particular illustrates the need of rigorous case selection of primary bladder and/or urachal adenocarcinomas in the studies. Inclusion of UC with glandular differentiation or plasmacytoid UC could significantly weaken the validity of such a study and therefore its conclusions.

The distribution of the DNA mismatch repair (MMR) proteins, that is, MutL homolog 1 (MLH1), MutS homolog 2 (MSH2), MutS homolog 6 (MSH6), and PMS1 homolog 2 (PMS2), in the different entities might in addition also be of differential diagnostic and pathogenetic interest. While no data is available for primary bladder adenocarcinomas, sporadic colorectal adenocarcinomas exhibit a loss of MMR proteins in 10–15% in total with emphasis on MLH1 [356]. In urachal adenocarcinomas, some tumors with microsatellite instability characterized by immunohistochemistry were described [25]. We, however, detected no loss of MMR proteins by immunohistochemistry in our own institutional cases (*n* = 12). In additional preliminary molecular analyses, we also did not detect evidence of microsatellite instability (unpublished data). This seems to point to molecular differences in adenocarcinomas of urachal and colorectal origin.

Further important biomarkers in differential diagnostic considerations of adenocarcinomas in general are hormone receptors. In our review data, urachal adenocarcinomas did not express estrogen and progesterone receptors by immunohistochemistry, which might be of particular interest in the discrimination of a metastasis of urachal adenocarcinomas to the ovary and vice versa [17, 56, 67]. In this setting, the immunonegativity of urachal adenocarcinomas for cancer antigen 125 (CA125) might also be of value, however, with the limitation of only 8 cases reported in the literature [27, 60, 86].

Low numbers of cases and no differential diagnostic value regarding the discrimination of urachal adenocarcinomas from primary bladder and colorectal adenocarcinomas were detected for *α*-fetoprotein (AFP), carbohydrate antigen 19-9 (CA19-9), cluster of differentiation 10 (CD10), CK19, Das-1, E48, E-Cadherin, gross cystic disease fluid protein 15 (GCDFP15), mucin 1 (epithelial membrane antigen) (MUC1 (EMA)), mucin 6 (MUC6), Thrombmodulin, thyroid transcription factor 1 (TTF1), Uroplakin III, Villin, and Vimentin [27, 48, 60, 86, 100, 106, 125, 126, 172, 198, 215, 217, 245, 292, 330].

However, this might not be the case for some rare and special differential diagnostic considerations as for example in the discrimination of ductal prostate cancer and the enteric type of urachal adenocarcinomas, in which immunohistochemistry for prostate specific acid phosphatase (PAP) and prostate-specific antigen (PSA) (both negative in urachal adenocarcinomas and positive in ductal prostate cancer) might be useful [21, 27, 74, 77, 100, 245, 255, 357].

In addition to the differential diagnostic context, immunohistochemical markers might be of further clinical value. We recently assessed the expression and prognostic relevance of six immunohistochemical markers (Ki67, p53, biglycan (BGN), receptor for hyaluronan-mediated motility (RHAMM), and insulin-like growth factor II mRNA binding protein 3 (IMP3)) in urachal adenocarcinomas. RHAMM, IMP3, Ki67, and p53 were found to be increased in urachal adenocarcinomas. However, none of the analyzed markers exhibited any prognostic information [15].

Although immunohistochemical biomarkers are widely used in differential diagnostic considerations, their interpretation is per se subjective. This applies in every situation in which these markers are used and therefore also in the immunohistochemical differential diagnosis of UrC. A further limitation of the collected data might also be the threshold at which the authors of the different source studies called an immunohistochemical marker positive or negative. Oftentimes, this information is missing while it can be very important. For example, the decision to call a case positive for nuclear *β*-Catenin might depend only on a few stained tumor nuclei but with important differential diagnostic implications [23, 329].

These considerations and also the partly overlapping positivity rates of the different immunohistochemical biomarkers make it difficult to recommend a step wise biomarker-guided approach. This could create a false sense of sensitivity and specificity of the biomarkers in the different situations. From our experience, immunohistochemical staining of a panel of antibodies, which depends on the differential diagnostic setting (Table 2), is the best way to come to a conclusion in this setting. This process might of course also include the use of further antibodies in addition to the core panel and always lies in the expertise of the diagnostic histopathologist.

## 8. Biomarkers in Urachal Cancer: Serum Markers

The (histomorphological) parallels between urachal and colorectal adenocarcinomas furthermore gave the rationale to test colorectal tumor markers in serum samples of patients with urachal adenocarcinomas, especially CEA, CA19-9, and CA125. In CRC, these markers are elevated in approximately one third (CA125), half (CA19-9), and two-thirds (CEA) of patients with a considerable variance depending on tumor size and other variables [358]. In primary bladder adenocarcinomas, however, only sporadic data is available with reports of elevated serum levels of these markers [359, 360].

In urachal adenocarcinomas, 44 studies including data on serum parameters were available, including 7 original studies and 37 case reports with a total of 140 patients.

Siefker-Radtke and colleagues reported on the largest cohort and found elevated (>3 ng/ml) CEA serum levels in 59% of patients with urachal adenocarcinomas (median: 36 ng/ml) [24]. In 5 cases, CEA also decreased in response to chemotherapy, suggesting the potential utility of CEA testing in monitoring (or follow-up) of UrC. When analyzing the literature, elevated CEA serum levels were reported in 55.7% (59/106) of patients at the time of diagnosis [24, 26, 33, 42, 60, 67, 79, 80, 86, 88, 89, 95, 99, 106, 118, 128, 131, 149, 156, 163–165, 167, 179, 196, 200, 207, 208, 212, 214, 240, 241, 244, 246, 251]. In our analyses, elevated CEA levels at diagnosis were associated with worse OS (*p* = 0.008) and PFS (*p* = 0.009) in dichotomized analyses (elevated versus normal), however, with only sparse survival data.

Additionally, elevated serum levels of CA19-9 and CA125 were reported in 50.8% (31/61) and 51.4% (19/37), respectively [24, 26, 33, 42, 76, 79–81, 86, 88, 89, 95, 99, 104, 114, 128, 149, 168, 200, 207, 213, 240, 244, 246, 251]. As with CEA, elevated levels of CA19-9 exhibited a trend towards worse OS and PFS (both *p* = 0.09). No prognostic association was noted for CA125. In addition, elevated serum levels of CA125 did not correlate with negative immunohistochemical tissue expression, however, with a low case number (*n* = 8).

Other serum biomarkers reported in low case numbers of urachal adenocarcinomas include lactate dehydrogenase (LDH) [80, 199], cancer antigen 15-3 (CA15-3) [26, 114, 156], AFP [16, 42, 95, 106, 156] with one case in a seven-month-old infant with a yolk sac tumor of the urachus [295], and neuron-specific enolase (NSE) [156] including one case of a neuroblastoma in a six-month-old child [296].

In summary, measurement of serum biomarkers might be useful in the follow-up and disease monitoring of UrC.

## 9. Conclusions

We identified a total of 1984 cases of UrC from 319 suitable studies with sufficient data from the English literature with overall 1834 adenocarcinomas (92%). While only minor variations in clinicopathological factors such as gender distribution (male preponderance), age at diagnosis, tumor size, and adenocarcinoma subtypes were noted, none of these factors were associated with overall survival. However, regarding progression-free survival, an advantage for male patients especially in the adenocarcinoma cohort was noted, while no such association was observed for nonglandular neoplasms of urachal origin.

The summary of existing evidence on immunohistochemical markers supplemented with our own data highlighted a differential diagnostic role for AMACR, CK34*β*E12, CK7, *β*-Catenin, CD15, and CEA (Table 2) which can be helpful in the routine differential diagnostic workup of adenocarcinomas in the bladder. Also, GATA3 might be helpful in the differentiation of urachal from primary bladder adenocarcinomas, with data presented almost exclusively derived from our institutional cohort. In addition, serum markers such as CEA, CA19-9, and CA125 might be useful in the follow-up and monitoring of UrC while CEA and CA19-9 may also be of prognostic value.

## Figures and Tables

**(a):** 

	*n*	%
Studies	319	100
Original study	72	22.6
Case report	235	73.7
Missing information	12	3.7
UrC total	1984	100
UrC adenocarcinoma total	1834	100
UrC adenocarcinoma specific type	1144	62.4
Mucinous	647	56.6
Enteric	172	15.0
NOS	157	13.7
Mixed	96	8.4
Signet ring	72	6.3
UrC adenocarcinoma type n/a	690	37.6
UrC nonadenocarcinoma	150	100
UrC nonadenocarcinoma specific type	124	82.7
UC	58	46.8
Sarcoma	34	27.4
SCC	26	21.0
Neuroendocrine npl.	6	4.8
UrC nonadenocarcinoma type n/a	26	17.3
Gender information available	1491	100
Male	936	62.8
Female	555	37.2
Age (mean/median) in years	48.6/50.8	
Tumor size (mean/median) in cm	7.1/6.0	

**(b):** 

	Overall survival			Progression-free survival		
	HR	95% CI	*p*	HR	95% CI	*p*
UrC type						
Adenocarcinoma	2.031	0.452–9.117	0.355	4.782	0.626–36.506	0.131
Nonadenocarcinoma	ref.			ref.		
UrC adenocarcinoma						
Mucinous	0.888	0.192–4.095	0.879	0.736	0.203–2.667	0.641
Nonmucinous	ref.			ref.		
UrC nonadenocarcinoma						
UC	n/a^∗^	n/a^∗^	n/a^∗^	n/a^∗^	n/a^∗^	n/a^∗^
Non-UC	ref.			ref.		
Gender						
Male	0.629	0.276–1.430	0.268	0.197	0.039–0.981	0.047
Female	ref.			ref.		
Age^^^						
<45 years	1.880	0.769–4.598	0.167	1.534	0.652–3.610	0.327
>45 years	ref.			ref.		
Tumor size^^^						
<7.0 cm	2.644	0.502–13.923	0.251	2.943	0.592–14.622	0.187
>7.0 cm	ref			ref.		

**Table 2:** Useful immunohistochemical antibodies in the differential diagnosis of urachal adenocarcinoma (UrC), colorectal adenocarcinoma (CRC), and primary bladder adenocarcinoma (PBAC). Loss of MMR proteins (MLH1, MSH2, MSH6, and PMS2) additionally favors colorectal over urachal adenocarcinomas. For more details on reactivity rates, number of cases, and references, please refer to Supplementary Table 2. Please note that data density is low for most antibodies limiting significance. “Highest” data quality is available for CK7, *β*-Catenin, and CEA.−: negative (0% positive); (−): mostly negative (1–25% positive); +/−: some positive (26–50% positive); (+): mostly positive (51–75% positive); +: positive (76–100% positive).

	Reactivity			Differential diagnosis		
Biomarker (IHC)	UrC	CRC	PBAC	UrC versus CRC and PBAC	UrC and PBAC versus CRC	UrC and CRC versus PBAC
AMACR (p504s)	(−)	+	(+)	+	−	−
CK34*β*E12 (HMWCK)	(+)	(−)	+/−	+	−	−
CK7	(+)	+/−	(+)	−	+	−
*β*-Catenin (nuclear)	(−)	+	(−)	−	+	−
CD15 (Leu-M1)	+	+/−	(+)	−	+	−
CEA	+	+	(+)	−	−	+
GATA3	−	−	(+)	−	−	+

## Supplementary Materials

Supplementary Materials Supplementary Table 1(a): Detailed information on used antibodies and protocol in the cohort of the UrC register of the University Hospital of Essen. Supplementary Table 1(b): IHC results in the analyzed cohort of the UrC register of the University Hospital of Essen Detection of any immunoreactivity in the tumor cells was assessed as positive and complete lack of immunoreactivity as negative. For *β*-Catenin, the nuclear (nuc.) and membranous/cytoplasmic (m/c) reactivities were analyzed separately. The DNA mismatch repair proteins MLH1, MSH2, MSH6, and PMS2 were evaluated as positive if more than 10% of tumor cells exhibited a nuclear immunoreactivity. Specific immunostaining was cytoplasmic in case of CD15, CEA, CK7, and CK20 and nuclear in case of CDX2 and GATA3. Supplementary Table 2: detailed information on immunohistochemical biomarker expression in urachal adenocarcinomas (UrC ADC) including own data (∗) in comparison to figures from the literature for colorectal (CRC) and primary bladder adenocarcinomas (PBAC). This table includes information about markers of potential differential diagnostic usefulness in UrC versus CRC and PBAC, UrC and PBAC versus CRC, and UrC and CRC versus PBAC. ^ is positive in hepatoid carcinomas of the urinary bladder [329]; ^^ is usually positive in clear cell PBAC [329]; ^^^Figures for sporadic colorectal cancer (nonhereditary), total loss of MMR proteins is evident in 10–15% of sporadic colorectal cancers [356] ^∗^Own data from the UrC register of the University Hospital of Essen is included to increase data quality (all: *n* = 12, but CK7: *n* = 11). [15] UrC: urachal cancer; ADC: adenocarcinoma; AFP: *α*-fetoprotein; AMACR: alpha-methylacyl-CoA racemase; mem/cyt: membranous/cytoplasmic immunoreactivity; *β*-Catenin: *β*-Catenin; CA19-9: carbohydrate antigen 19-9; CA125: cancer antigen 125; CD10: cluster of differentiation 10; CD15: cluster of differentiation 15; CDX2: caudal-type homeobox protein 2; CEA: carcinoembryonic antigen; CK7: cytokeratin 7; CK19: cytokeratin 19; CK20: cytokeratin 20; CK34*β*E12 (HMWCK): cytokeratin 34 (1,5,10,14) (high-molecular weight cytokeratin); ER: estrogen receptor; GATA3: GATA binding protein 3; GCDFP15: Gross cystic disease fluid protein 15; HER2: human epidermal growth factor receptor 2; MMR: DNA mismatch repair proteins; MLH1: mutL homolog 1; MSH2: mutS homolog 2; MSH6: mutS homolog 6; MUC1 (EMA): mucin 1, cell surface associated (epithelial membrane antigen); MUC2: mucin 2, oligomeric mucus/gel-forming; MUC5AC: mucin 5AC oligomeric mucus/gel-forming; MUC6: mucin 6, oligomeric mucus/gel-forming; PR: progesterone receptor; PSAP: prostatic specific acid phosphatase; PSA: prostate-specific antigen; PMS2: PMS1 homolog 2; RegIV: Regenerating gene IV; TTF1: thyroid transcription factor 1 (NK2 homeobox 1). Supplementary Figure 1: progression-free survival in urachal adenocarcinomas regarding gender [1, 15, 17, 21, 23, 25–27, 31, 36, 48, 56, 60, 62, 67, 74, 77, 86, 95, 99, 100, 106, 124–126, 128, 148, 156, 163–165, 172, 198, 215–217, 220, 221, 240, 245, 251, 255, 292, 327–335, 338–355, 361–388].
