# Supplementary material for: Biomarkers in Urachal Cancer and Adenocarcinomas in the Bladder: A Comprehensive Review Supplemented by Own Data
Source: Dis Markers. 2018 Mar 12;2018:7308168. doi: 10.1155/2018/7308168 (PMC5867586; doi:10.1155/2018/7308168)
Supplement: Supplementary Materials — Supplementary Table 1(a): Detailed information on used antibodies and protocol in the cohort of the UrC register of the University Hospital of Essen. Supplementary Table 1(b): IHC results in the analyzed cohort of the UrC register of the University Hospital of Essen Detection of any immunoreactivity in the tumor cells was assessed as positive and complete lack of immunoreactivity as negative. For β-Catenin, the nuclear (nuc.) and membranous/cytoplasmic (m/c) reactivities were analyzed separately. The DNA mismatch repair proteins MLH1, MSH2, MSH6, and PMS2 were evaluated as positive if more than 10% of tumor cells exhibited a nuclear immunoreactivity. Specific immunostaining was cytoplasmic in case of CD15, CEA, CK7, and CK20 and nuclear in case of CDX2 and GATA3. Supplementary Table 2: detailed information on immunohistochemical biomarker expression in urachal adenocarcinomas (UrC ADC) including own data (∗) in comparison to figures from the literature for colorectal (CRC) and primary bladder adenocarcinomas (PBAC). This table includes information about markers of potential differential diagnostic usefulness in UrC versus CRC and PBAC, UrC and PBAC versus CRC, and UrC and CRC versus PBAC. ^ is positive in hepatoid carcinomas of the urinary bladder [329]; ^^ is usually positive in clear cell PBAC [329]; ^^^Figures for sporadic colorectal cancer (nonhereditary), total loss of MMR proteins is evident in 10–15% of sporadic colorectal cancers [356] ∗Own data from the UrC register of the University Hospital of Essen is included to increase data quality (all: n = 12, but CK7: n = 11). [15] UrC: urachal cancer; ADC: adenocarcinoma; AFP: α-fetoprotein; AMACR: alpha-methylacyl-CoA racemase; mem/cyt: membranous/cytoplasmic immunoreactivity; β-Catenin: β-Catenin; CA19-9: carbohydrate antigen 19-9; CA125: cancer antigen 125; CD10: cluster of differentiation 10; CD15: cluster of differentiation 15; CDX2: caudal-type homeobox protein 2; CEA: carcinoembryonic antigen; CK7: cytokera [file 7308168.f1.pdf]

Antibody	Company	Clone/#	Clonality	Dilution	Incubation	Detection
**bCatenin**	BDTransduction	610154	monoclonal	1:2000	32min, 37°C	OptiView Ventana, DAB
**CD15**	Ventana	MMA	monoclonal	ready to use	16min, 37°C	OptiView Ventana, DAB
**CDX2**	Zytomed	EPR2764Y	monoclonal	1:400	24min, 37°C	OptiView Ventana, DAB
**CEA**	Dako	A0115	polyclonal	1:12000	24min, 37°C	OptiView Ventana, DAB
**CK7**	Dako	OV-TL 12/30	monoclonal	1:5000	16min, 37°C	OptiView Ventana, DAB
**CK20**	Dako	Ks20.8	monoclonal	1:1000	24min, 37°C	OptiView Ventana, DAB
**GATA3**	Santa Cruz	HG3-31	monoclonal	1:30	40min, 37°C	OptiView Ventana, DAB
**MLH1**	Zytomed	G168-15	monoclonal	1:75	60min, 37°C	OptiView Ventana, DAB
**MSH2**	DCS	25D12	monoclonal	1:100	20min, 37°C	OptiView Ventana, DAB
**MSH6**	DCS	44	monoclonal	1:400	24min, 37°C	OptiView Ventana, DAB
**PMS2**	Dako	M3647	monoclonal	1:25	60min, 40°C	OptiView Ventana, DAB

Supplementary Table 1a) Detailed information on used antibodies and protocol in the cohort of the UrC register of the University Hospital of Essen

Biomarker	pos. (%)	neg. (%)
**bCatenin nuc.**	6 (50%)	6 (50%)
**bCatenin m/c**	12 (100%)	0 (0%)
**CD15**	9 (75%)	3 (25%)
**CDX2**	11 (92%)	1 (8%)
**CEA**	12 (100%)	0 (0%)
**CK7**	6 (55%)	5 (45%)
**CK20**	12 (100%)	0 (0%)
**GATA3**	0 (0%)	12 (100%)
**MLH1**	12 (100%)	0 (0%)
**MSH2**	12 (100%)	0 (0%)
**MSH6**	12 (100%)	0 (0%)
**PMS2**	12 (100%)	0 (0%)

Supplementary Table 1b) IHC results in the analyzed cohort of the UrC register of the University Hospital of Essen
Detection of any immunoreactivity in the tumor cells was assessed as positive and complete lack of immunoreactivity as negative. For β-Catenin the nuclear (nuc.) and membranous/cytoplasmic (m/c) reactivities were analyzed separately. The DNA mismatch repair proteins MLH1, MSH2, MSH6, and PMS2 were evaluated as positive if more than 10% of tumor cells exhibited a nuclear immunoreactivity. Specific immunostaining was cytoplasmic in case of CD15, CEA, CK7, and CK20 and nuclear in case of CDX2 and GATA3.

Supplementary Table 2	UrC ADC total			CRC	PBAC	UrC vs. CRC& PBAC	UrC& PBAC vs. CRC	UrC &CRC vs. PBAC	References UrC ADC	References CRC/PBAC
**Biomarker (IHC)**	n	pos / neg	positive	positive	positive					
AFP	1	1 / 0	100%	12%	0%^				[106]	[351, 361]
AMACR (p504s)	12	2 / 10	17%	65-76%	65%	yes			[17, 77]	[17, 343, 344]
β-Catenin nuclear *	63	9 / 54	14%	64-95%	0-17%		yes		[1, 17, 23, 26, 125, 216, 220, 328]	[23, 329, 335, 338, 354, 362]
β-Catenin mem/cyt *	65	64 / 1	98%	74-100%	78-88%				[1, 23, 26, 125, 216, 220, 328]	[221, 338, 362]
CA19-9	1	1 / 0	100%	42-100%	-				[86]	[352, 353, 363]
CA125	8	0 / 8	0%	0-64%	28%^^				[27, 60, 86]	[27, 352, 364-366]
CD10	1	1 / 0	100%	36-56%	55%				[100]	[335, 367, 368]
CD15 (Leu-M1) *	21	18 / 3	86%	44%	73%		yes		[21, 124, 125]	[21, 27, 348]
CDX2 *	96	86 / 10	90%	83-99%	13-83%				[1, 17, 23, 26, 31, 60, 99, 125, 126, 128, 216, 217, 240, 245, 251, 327, 330]	[23, 329-335, 344, 366]
CEA *	38	38 / 0	100%	93-100%	29-67%			yes	[21, 27, 48, 60, 67, 77, 86, 100, 106, 124, 163, 165, 245, 255, 349, 350]	[21, 27, 348, 351-353]
CK7 *	100	51 / 49	51%	0-38%	33-70%		yes		[1, 17, 23, 26, 27, 31, 36, 48, 56, 60, 67, 74, 77, 86, 99, 124, 125, 156, 163, 164, 216, 217, 220, 240, 251, 255, 292, 327, 328]	[23, 27, 329, 335, 338, 344, 346]
CK19	1	1 / 0	100%	58-96%	-				[126]	[346, 369]
CK20 *	116	112 / 4	97%	68-96%	53-100%				[1, 17, 23, 26, 27, 31, 36, 48, 56, 60, 62, 67, 74, 77, 86, 95, 99, 124-126, 128, 148, 156, 163, 164, 216, 217, 220, 240, 245, 251, 327]	[27, 329, 335, 338, 344, 346, 366, 370]

CK34βE12 (HMWCK)	18	12 / 6	67%	11-17%	variable	yes			[1, 26, 77, 125, 255]	[1, 345]
Claudin18	34	18 / 16	53%	<5%	-				[23]	[23, 339-341]
Das-1	6	6 / 0	100%	65- 77%	-				[172, 198]	[371, 372]
E48	5	1 / 4	20%	23%	3%				[27]	[27]
ECadherine	1	0 / 1	0%	56-100%	91%				[86]	[335, 373]
ER	13	0 / 13	0%	0-2%	0%				[17, 56, 67]	[352, 361, 366]
GATA3 *	14	0 / 14	0%	0%	0-56%			yes	[355]	[335, 354, 355]
GCDFP15	2	0 / 2	0%	0-9%	-				[60]	[352, 366]
HER2	6	0 / 6	0%	5-7%	0-5%				[27, 56]	[27, 361, 374, 375]
MLH1 (MMR) *	19	19 / 0	100%	74-96%^^^	-				[25]	[376-378]
MSH2 (MMR) *	19	18 / 1	95%	94-98%^^^	-				[25]	[376-378]
MSH6 (MMR) *	19	18 / 1	95%	95-99%^^^	-				[25]	[378, 379]
MUC1 (EMA)	5	4 / 1	80%	25-83%	58%				[48, 86, 125, 217, 245]	[346, 353, 380, 381]
MUC2	13	13 / 0	100%	33-82%	60%	maybe			[86, 100, 125, 128, 251]	[335, 346, 347]
MUC5AC	12	11 / 1	92%	6-49%	60%	maybe			[86, 95, 125, 128]	[335, 346, 347]
MUC6	2	0 / 2	0%	4-39%	0%				[86, 125]	[346, 347, 361]
p63	34	1 / 33	3%	0%	0-18%				[23, 292]	[335, 353, 361]
PR	13	0 / 13	0%	0%	0%				[17, 56, 67]	[345, 361, 382]
PSAP	13	0 / 13	0%	0%	0%				[21, 27]	[27, 345, 383, 384]
PSA	17	2 / 15	12%	0%	0%				[21, 27, 74, 77, 100, 245, 255]	[27, 345, 366, 383, 384]
PMS2 (MMR) *	19	17/ 2	89%	98%^^^	-				[25]	[378]
RegIV	34	29 / 5	85%	36-100%	-				[23]	[23, 342]
Thrombmodulin	2	0 / 2	0%	0-27%	59%				[60]	[338, 385]
TTF1	3	0 / 3	0%	0-3%	-				[60, 215]	[353, 366, 386-388]
Uroplakin III	2	0 / 2	0%	0%	9%				[60, 292]	[335]
Villin	2	0 / 2	0%	67-100%	65-100%				[60, 330]	[329, 343-345]
Vimentin	6	0 / 6	0%	0%	0%				[27, 217]	[27, 361]

Supplementary Table 2) Detailed information on immunohistochemical biomarker expression in urachal adenocarcinomas (UrC ADC) including own data (*) in comparison to figures from the literature for colorectal (CRC) and primary bladder adenocarcinomas (PBAC)
This table includes information about markers of potential differential diagnostic usefulness in UrC vs. CRC&PBAC, UrC & PBAC vs. CRC and UrC & CRC vs. PBAC.

^: is positive in hepatoid carcinomas of the urinary bladder [329]; ^^: is usually positive in clear cell PBAC [329]; ^^^: figures for sporadic colorectal cancer (non-hereditary), total loss of MMR proteins is evident in 10-15% of sporadic colorectal cancers [356]
*: own data from the UrC register of the University Hospital of Essen is included to increase data quality (all: n=12, but CK7: n=11). [15]

UrC: urachal cancer, ADC: adenocarcinoma, AFP: α-fetoprotein, AMACR: alpha-methylacyl-CoA racemase, mem/cyt: membranous/cytoplasmic immunoreactivity, β-Catenin: β-Catenin; CA19-9: carbohydrate antigen 19-9, CA125: cancer antigen 125, CD10: cluster of differentiation 10, CD15: cluster of differentiation 15, CDX2: caudal-type homeobox protein 2, CEA: carcinoembryonic antigen, CK7: cytokeratin 7, CK19: cytokeratin 19, CK20: cytokeratin 20, CK34βE12 (HMWCK): cytokeratin 34 (1,5,10,14) (high-molecular weight cytokeratin), ER: estrogen receptor, GATA3: GATA binding protein 3, GCDFP15: Gross cystic disease fluid protein 15, HER2: human epidermal growth factor receptor 2, MMR: DNA mismatch repair proteins, MLH1: mutL homolog 1, MSH2: mutS homolog 2, MSH6: mutS homolog 6, MUC1 (EMA): mucin 1, cell surface associated (epithelial membrane antigen), MUC2: mucin 2, oligomeric mucus/gel-forming, MUC5AC: mucin 5AC, oligomeric mucus/gel-forming, MUC6: mucin 6, oligomeric mucus/gel-forming, PR: progesterone receptor, PSAP: prostatic specific acid phosphatase, PSA: Prostate-specific antigen, PMS2: PMS1 homolog 2, RegIV: Regenerating gene IV, TTF1: thyroid transcription factor 1 (NK2 homeobox 1)

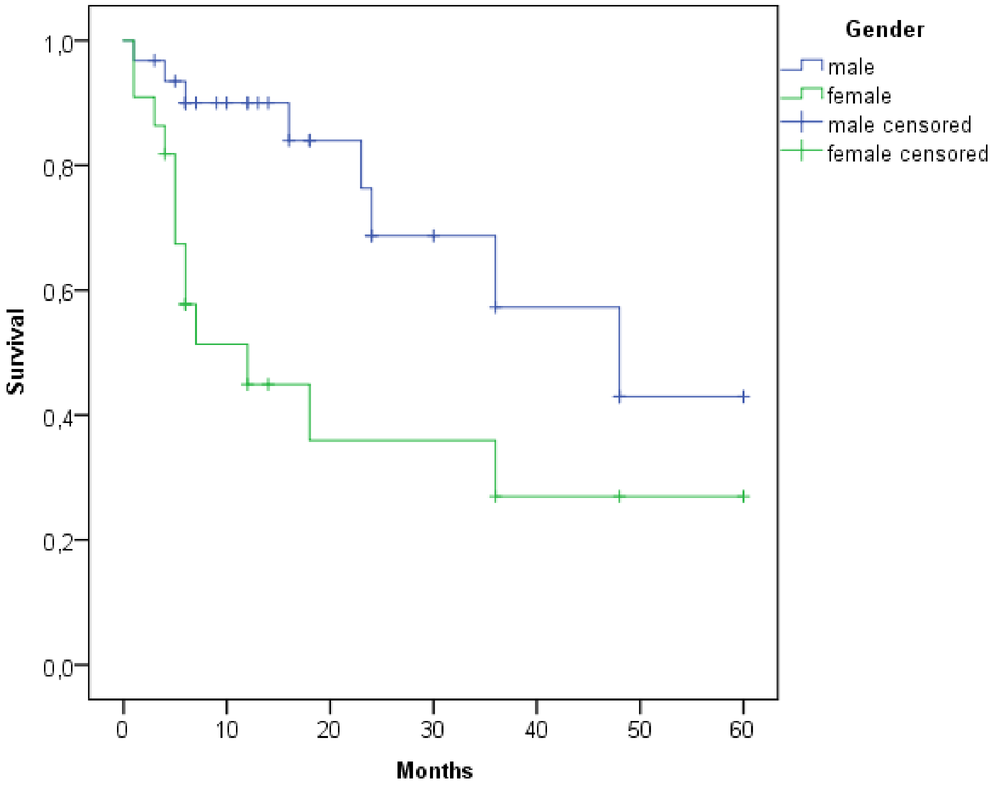

Supplementary Figure 1) Progression-free survival in urachal adenocarcinomas regarding gender
